# JDart: Portfolio Solving, Breadth-First Search and SMT-Lib Strings (Competition Contribution)

**DOI:** 10.1007/978-3-030-72013-1_30

**Published:** 2021-02-26

**Authors:** Malte Mues, Falk Howar

**Affiliations:** 8 Eindhoven University of Technology, Eindhoven, The Netherlands; 9 Aalborg University, Aalborg East, Denmark; TU Dortmund University, Dortmund, Germany

## Abstract

JDart performs dynamic symbolic execution of Java programs: it executes programs with concrete inputs while recording symbolic constraints on executed program paths. A portfolio of constraint solvers is then used for generating new concrete values from recorded constraints that drive execution along previously unexplored paths. For SV-COMP 2021, we improved JDart by implementing exploration strategies, bounded analysis, and path-specific constraint solving strategies, as well as by enabling the use of SMT-Lib string theory for encoding of string operations.

## 1 Overview

JDart is a dynamic symbolic execution engine for the Java virtual machine (JVM) built on top of Java PathFinder (JPF) [12]. We first entered SV-COMP 2020 with JDart. Our corresponding report gives a short overview of JDart's architecture and internals [9]. In this paper, we focus on the description of the following three improvements that were explicitly motivated by SV-COMP 2021 [2].

1. The re-implementation of the internal constraints-tree enables bounded analysis and exploration strategies (e.g., breadth first search instead of depth first search),
2. A new CVC4 backend in JConstraints is the basis for path-based selection of constraint solvers and sequential portfolio solving (using Z3 and CVC4).
3. We integrate recent advances in string constraint solving [3,10] by modeling string operations as SMT-Lib string constraints instead of bit vectors.

While all three changes contribute to an improved performance of JDart, portfolio solving has by far the biggest impact on the number of analyzed benchmark instances of SV-COMP 2021. In this paper, we focus on the description of the changes for (1) and (2).

## 2 Tool Improvements for SV-COMP 2021

JDart runs as an extension of the JPF software model checker [12], using the Java virtual machine implemented by JPF and its capabilities for annotating

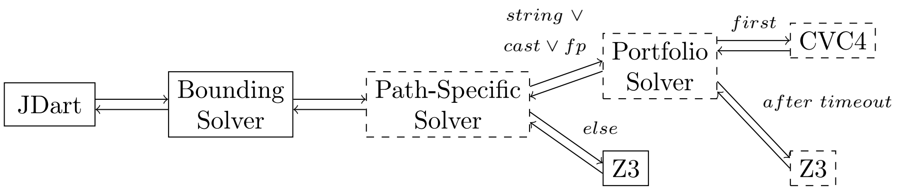

Fig. 1: The architecture and call hierarchy in the constraint solving backend.

values on the stack and the heap with symbolic information. The tool itself is written in Java and uses JConstraints [6] for encoding SMT problems. Moreover, JConstraints acts as a frontend to the Z3 [5] or CVC4 [1] SMT solver used for finding concrete values that drive the analysis.

**Exploration Strategies.** JDart has two main components: the *Executor* and the *Explorer*. While the *Executor* runs the concrete analysis and records symbolic constraints during concrete execution, the *Explorer* is responsible for exploration strategies and management of constraints. We re-designed the central data structure of the *Explorer*, the constraints tree, for SV-COMP 2021: The new tree supports different exploration strategies (e.g., breadth-first search) and bounds on the depth of exploration. In the past, JDart relied on unbounded depth-first exploration which would often 'get trapped' unrolling unbounded loops or recursion. Breadth-first search prevents this behavior and is more effective on the SV-COMP benchmark set.

**Portfolio-Solving.** Figure 1 demonstrates the architecture of the constraint solving backend used by JDart and JConstraints for SV-COMP; dashed components and control-flow have been added for SV-COMP 2021: The *bounding solver* (developed for SV-COMP 2020) calls subsequent solvers with successively weaker bounds on numeric variables. For SV-COMP 2021, we use upper bounds 2, 8, 13, 21, 200, 600, $\infty$ and symmetric negative lower bounds. The new *path-specific solver* selects the most promising solving approach for every concrete path constraint: Currently, constraints involving string operations, type casts, or floating-point numbers are handed to the portfolio solver as we expect better performance. The *portfolio solver* wraps the CVC4 solver, starting repeated solving attempts in the case of (fairly frequent and random) segmentation faults as well as invocation of Z3 after a fixed timeout of 60 seconds. All other path constraints are passed directly to the Z3 solver as JDart used to do with all constraints at SV-COMP 2020.

## 3 Strengths and Weaknesses

JDart scored 623 points (max. of 693) in the Java track and was declared second winner for Java, after Java Ranger (630 points) [11]. Next best is JBMC [4] with 603 points. As Java Ranger and JBMC, JDart did not report a single incorrect verdict. JDart exhibits the general strengths and weaknesses of dynamic and symbolic analysis approaches for Java programs:

**Fast search for counterexamples.** Driven by concrete execution, the analysis is fairly fast. JDart ($950s$)is overall the second fastest tool in cases where it can provide an answer after JBMC ($650s$). Notably, JDart successfully found counterexamples in 251 of 253 instances. The second-best tool in this respect is JBMC with 243 correct *false* verdicts. Of the two instances for which JDart did not produce counterexamples one uses the `split` operation for strings that JDart does not yet model, leading to an *unknown* result. For the other instance, stack unrolling triggers an out of memory exception during the concolic execution of one path through the recursive Ackermann function.

**Path Explosion.** JDart is affected by path explosion in programs with long sequences of branching instructions with mutually unrelated conditions. Such sequences are common in code generated from models in the realm of embedded systems, e.g., by the *Alarm* benchmark instances in SV-COMP 2021. For these instances, JDart does not manage to explore all paths in the given time limit.

**Unbounded Behavior.** Based on principles of symbolic execution, JDart will only terminate on unbounded loops or in case of unbounded recursion when using manually configured bounds. In addition, the concolic execution might be configured to stop on property violations. As a consequence, assertion errors might be used as analysis bounds. For SV-COMP 2021, we used a search depth of 270 recorded decisions on paths in the constraints tree which we deemed conservative after initial experiments on the benchmark set: While in 13 instances *true* verdicts were given after exploring exhaustively up to the depth bound, there remain 30 problem instances for which JDart timed out exploring the search space up to the depth bound and 6 instances raising *unknown* verdicts (including the two mentioned above).

## 4 Tool Setup

The source code of JDart used for the competition artifact [8] is available on GitHub[1]. JDart is designed as a plug-in for JPF and relies on ant as a build system. One of its dependencies is the `jpf-core` project [12]. The other dependency is the JConstraints library, which was configured to use Z3 [5] and CVC4 [1] for SV-COMP 2021. For the competition, JDart is wrapped by the `run-jdart.sh` shell script which generates `.jpf` configuration files, specifying which benchmark to analyze and the global configuration options of JDart. For SV-COMP 2021, we choose termination on the first assertion error, a depth bound of 270 (decisions on paths in the constraints tree) for exploration, breadth first search as exploration strategy, and the described path-specific solver together with iterative weakening of bounds on values in models as described in Section 2. Z3 is configured to run with the sequence solver for strings. The shell script records and interprets the output of JDart and can also report the version of JDart.

[1] https://github.com/tudo-aqua/jdart, Commit `4a9cc43`

## 5 Software Project

JDART, as used in SV-COMP 2021, is maintained by the Automated Quality Assurance Group at TU Dortmund University (in particular by the authors of this paper) and is available under the Apache License, version 2.0, on GitHub[1]. An initial version of JDART was developed by the authors of [7] at NASA Ames Research Center and Carnegie Mellon University. The original version of JDART is available on GitHub[2].
